# A Framework for Sharing and Integrating Remote Sensing and GIS Models Based on Web Service

**DOI:** 10.1155/2014/354919

**Published:** 2014-05-11

**Authors:** Zeqiang Chen, Hui Lin, Min Chen, Deer Liu, Ying Bao, Yulin Ding

**Affiliations:** ^1^Institute of Space and Earth Information Science, The Chinese University of Hong Kong, Shatin, Hong Kong; ^2^Collaborative Innovation Center for Geospatial Information Technology, Wuhan University, Wuhan 430079, China; ^3^Shenzhen Research Institute, The Chinese University of Hong Kong, Shenzhen 518057, China; ^4^Department of Geography and Resource Management, The Chinese University of Hong Kong, Shatin, Hong Kong; ^5^Faculty of Architectural and Survey Engineering, Jiangxi University of Science and Technology, Ganzhou 341000, China; ^6^International Institute for Earth System Science, Nanjing University, Nanjing 210023, China; ^7^State Key Laboratory of Information Engineering in Surveying, Mapping and Remote Sensing, Wuhan University, Wuhan 430079, China

## Abstract

Sharing and integrating Remote Sensing (RS) and Geographic Information System/Science (GIS) models are critical for developing practical application systems. Facilitating model sharing and model integration is a problem for model publishers and model users, respectively. To address this problem, a framework based on a Web service for sharing and integrating RS and GIS models is proposed in this paper. The fundamental idea of the framework is to publish heterogeneous RS and GIS models into standard Web services for sharing and interoperation and then to integrate the RS and GIS models using Web services. For the former, a “black box” and a visual method are employed to facilitate the publishing of the models as Web services. For the latter, model integration based on the geospatial workflow and semantic supported marching method is introduced. Under this framework, model sharing and integration is applied for developing the Pearl River Delta water environment monitoring system. The results show that the framework can facilitate model sharing and model integration for model publishers and model users.

## 1. Introduction

Sharing and integrating Remote Sensing (RS) and Geographic Information System/Science (GIS) models are important for solving comprehensive, complex, and multidisciplinary problems, such as urban growth [1] and environmental applications [2, 3]. However, with the inherent natural characteristics, such as heterogeneity (e.g., structure and execution environment) and dispersion (e.g., owner and running server), and the man-made characteristics, such as privacy (e.g., authorization-required and classified), of RS and GIS models, simple and effective sharing and integrating RS and GIS models are still challenges, although many efforts have been made to improve model sharing and integration strategies [4–6].

Recent studies have focused on model sharing and integration problems [7, 8]. Decades ago, a number of model representation approaches and languages, such as algebraic modeling languages [9–11], logic-based systems [12], relational-based approaches [13, 14], graph-based languages [4, 15–18], and structured modeling [19, 20], were proposed to handle model sharing and integration issues by providing a consistent representation method, but they have limited support for model sharing in a distributed environment [21]. Distributed object technologies have been employed to access remote objects in a distributed environment, such as the Common Object Request Broker Architecture (CORBA) [22], the Distributed Component Object Model (DCOM) [23], and the Java Remote Method Invocation (Java RMI) [24]; the direct use of these three technologies by programmers is becoming less common for their shortcomings: tightly coupled protocols are effective for building a specific application but are not sufficiently flexible, and protocols are constrained by their vendor implementations, platforms, and languages [25].

In recent years, standard technologies, specifications, and frameworks have been developed for model sharing and integration; examples include the Model Driven Architecture (MDA) and the open modeling interface (OpenMI) as well as several modeling frameworks. MDA is a software design approach developed by the Object Management Group to provide an open and vendor-neutral approach for software systems using specifications such as The Unified Modeling Language, the Meta Object Facility, the XML Metadata Interchange, and the Common Warehouse Meta-model that built coherent schemes for authoring, publishing, and managing models with the platform independent model PIM and the platform-specific model PSM [26, 27]. The MDA is promising, but the PIM and the PSM do not include standard specification mapping, which complicates application of the MDA. The open modeling interface (OpenMI) is a software component interface that allows time-dependent models to share and exchange information at runtime, making model integration feasible at the operational level. OpenMI emphasizes interoperability between otherwise independent models [28]. Dozens of modeling and reuse frameworks, such as the spatial modelling environment (SME) [29, 30] and the interactive component modelling system (ICMS) [31], have been developed to handle model sharing and integration problems, but most of them are highly field-related, platform-related, and internet-inaccessible, which limits their application [5].

With the development of information technology (IT), Web service and service-oriented architecture (SOA) are more commonly used in model sharing and model integration [2, 32–34]. Some advantages include sharing models as worldwide services applied to various popular applications [35] and integrating data and models from anywhere in the world [36]. In addition, with the advent of the advanced notion of “Model as a Service” [37–40], the Web service method creates new opportunities for sharing and integrating models, including GIS and RS models.

The objective of this paper is to design a Web service-based method to share and integrate RS and GIS models. The main idea of model sharing and integration based on Web service is to release various heterogeneous RS and GIS models as Web services for model sharing and then to integrate the homogeneous Web services for model integration. Following this idea, this paper publishes the methods model and the model integration as Web services and applies this to the water environment monitoring in the Pearl River Delta region in China.

The organization of this paper is as follows: Section 2 addresses the main idea of web service-based model sharing and integration and the proposed method; Section 3 describes the implementation of the proposed method for the PRD water environment monitoring system. Finally, Section 4 concludes this paper and outlines future work.

## 2. Methodologies

A model is a representation of one or more processes that are believed to occur in the real word [41]. From a user's perspective, a model can be formalized as a tuple as *M* = {ID, *N*, *I*, *O*, IMPL, MD}, where *M* is the model, ID presents the unique identification of the model, *N* is its name, and *I*, *O*, IMPL, and MD are the input, the output, the implementation (executable programs), and the metadata of the model, respectively.

**Figure 1:**
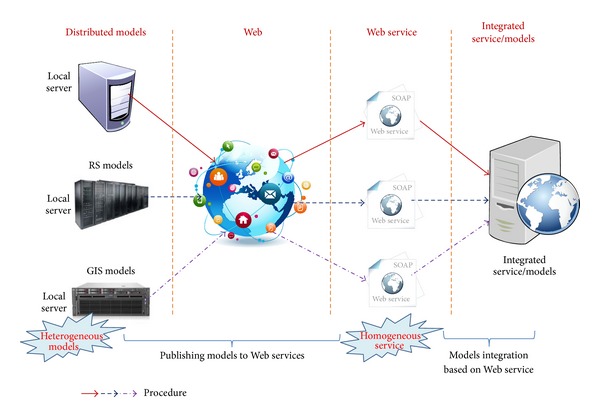
The framework of GIS and RS model sharing and integration based on Web service.

Web service offers a way to share and interoperate models [40]. The method is based on Simple Object Access Protocol (SOAP) and Web Services Description Language (WSDL). WSDL provides a function-centric description of the Web services, including their inputs, outputs, and exception handing [42], which map to the structure of the formalized model tuple. Because the method is standardized and loosely coupled [40], heterogeneous RS and GIS models become “homogeneous” Web services after they are published. Integrating heterogeneous RS and GIS models is equivalent to integrating the “homogeneous” Web services because the models are published as Web services. Web service composition is a method of integrating Web services [33, 34, 43]. Web service composition aggregates multiple services into a single new service for a certain functionality that a single primary service cannot provide [44]. If a model that runs on a desktop or local area network environment is called the local model and a model that is published with Web service is called a Web model, the aim of sharing and integrating the GIS and RS models is to publish the local model to a Web model and then combine the Web models. The framework of sharing and integrating GIS and RS models based on Web service is shown in Figure 1.

### 2.1. Model Sharing with Web Service

**Figure 2:**
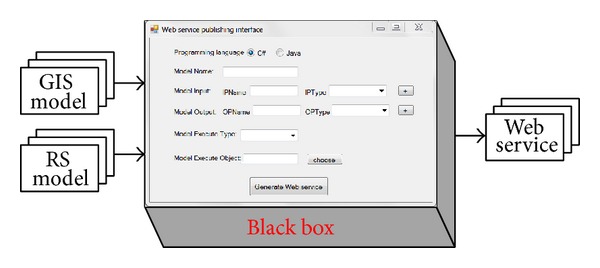
The black box approach combined with a visual method for publishing local models to Web services.

Model sharing is the publishing of local models as Web models using a Web service. Developing a Web service is time intensive and requires specific professional skills. In addition, many GIS and RS model experts aim to concentrate their efforts on developing more powerful models rather than on improving their programming skills. Considering these factors, a black box approach combined with a visual method is introduced to facilitate the publishing of local models to Web services. A black box [45] is a method that can be used without knowing how its inner algorithm works; the user only needs to know the input and output characteristics. The exposed entry of the black box is a visual user interface that is illustrated in Figure 2. A visual method for manipulating a model is an effective way to reduce development time and training [46]. The visual interface hides the cumbersome implementation of the Web service.

For this method, “inside” the black box is critical, generating Web services from GIS and RS models with their names, inputs, outputs, and executable programs. The inputs to the black box include description parameters, and the output includes the Web functions. An interface method is introduced to perform these functions. Interface-based programming is a common programming method in high-level programming languages. An interface is a reference-type object that defines methods without defining the implementation. It is a signature for interacting with other classes or interfaces. An interface-based method separates a method definition and its implementation and makes code more reusable, robust, revisable, and abstract. The purposes of adopting an interface-based method are (1) to provide a uniform method of describing the inputs and outputs for a model “outside” the black box, which facilitates the interaction between a model and the black box and (2) to make the Web service implementation easy with a unified interface “inside” the black box. Two important items for the interface-based method are the interface definition and the interface implementation.

**Figure 3:**
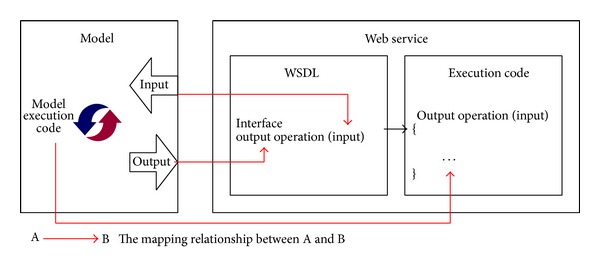
Mapping a model to a Web service.

For the interface definition, the key of publishing a model to a Web service is mapping the description structures between the model and the Web service. The* Operation* in a WSDL document is similar to a method or function in a traditional programming language. An executable model is also similar to a method or function in a traditional programming language. The outline for mapping the relationship between a model and a Web service is shown in Figure 3. The input, output, and execution code of a model map to the input, output, and execution code of a Web service, respectively. The execution code of the model is its programming implementation, which is consistent with the Web service.

In Figure 2, the name, input, and output of a model map to the name, input, and output of an interface, respectively. The input and output parameters are set with names and types. The types are basic programming types, such as* string*,* double,* and* int*. These parameter names and types are consistent with those of the model executable program inputs and outputs.

For interface implementation, models deployed in a local server may occur in one of many forms, for example, executable file (EXE), script, dynamic link library (DLL), or other written program languages. These form-executable programs are called third-party programs. Invoking a third-party program is essential to run a local model. High-level programming languages are able to invoke third-party programs. For instance, Java uses runtime and process classes to run third-party programs, and in C#, DllImport is used to execute third-party programs. A Web service development library supports the development of Web services. C# includes Web service classes in the  .NET Framework as library classes for the development of Web services. Java also has library classes for Web service development. Java has many open source projects such as Axis2 that can be used to develop and deploy Web services. In Figure 2, a Web service skeleton is shown for the interface programming method that maps a model to a Web service. When a concrete interface is set, the third-party program fills in the skeleton to realize the Web service.

### 2.2. Model Integration Using a Web Service

RS and GIS models are homogeneous Web services after the models are published into Web models. The model integration challenge is equivalent to Web service integration. Web service integration consists of combining different but associated Web services on the Web. A Web service demonstrates its capabilities by WSDL with operations. Composing Web services includes combining operations using a logical process. Web service integration creates an order-logic combination of related Web services.

Web service composition has been studied for years. Industry and academia each have presented numerous Web services composition methods. Web service composition methods can be divided into workflow-based service compositions and artificial intelligence-based (AI) service compositions in accordance with their technical and theoretical bases. For the workflow method, a composite service is similar to a workflow that contains a set of Web services together with the control and data flow among the services (e.g., [33, 34, 46, 47]). For the AI method, the Web service composition can be regarded as an automatic method of finding the solution to a planning problem: given an initial state and the target state, seek a path to achieve the service portfolio from the initial state to the target state in a collection of services (e.g., [48–50]). The automatic and intelligent AI-based Web service composition method develops the trend and the final purpose; however, the workflow method is more mature in industry. In this paper, a Web service composition framework based on both of these methods is proposed.

**Figure 4:**
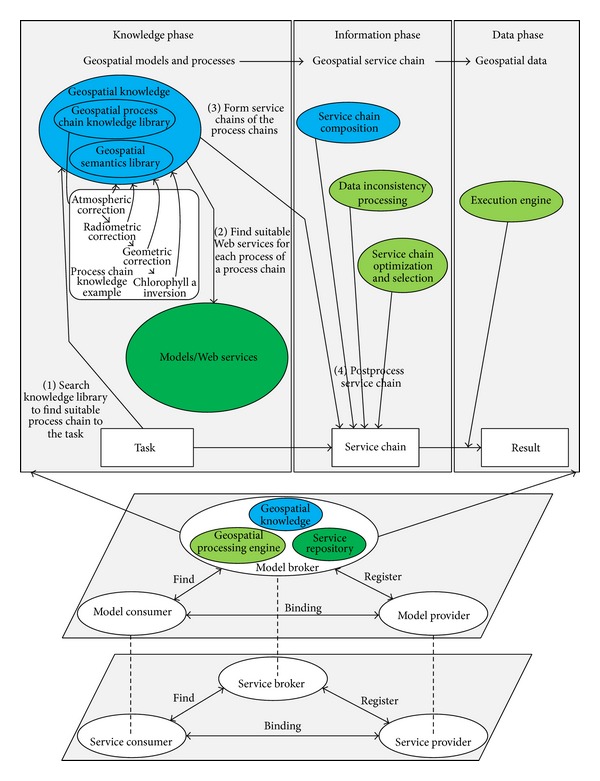
Integrate GIS and RS models based on Web service under the GPW framework.

Chen et al. [33, 34] introduced a geospatial processing workflow (GPW) method to integrate geo-related Web services for a complex task; this method has some notable advantages over other methods: interoperability, flexibility, and reusability. The GPW method provides a general framework called the abstract GPW, which defines the conception process of a task and instantiates a concrete GPW in a specific application. Abstract GPW consists of three phases: knowledge, information, and data [33]. The knowledge phase defines geospatial models and processes. The information phase integrates geospatial processes into a geospatial service chain. The data phase executes a geospatial service chain to generate data. This paper focuses on the GPW method for GIS and RS Web service composition. A framework for integrating GIS and RS models based on Web service using the GPW method is proposed as depicted in Figure 4.

Analogous with the roles in SOA, there are three model roles in the framework shown in Figure 4: the model provider, the model broker, and the model consumer.

- The model provider publishes models to Web services (described in Section 2.1) and registers them with the model broker.
- The model consumer is a user who finds a solution for a task from the service broker.
- The model broker is a model metainformation repository and a task solver.

Core parts in the model broker are the geospatial knowledge, the service repository, and the geospatial processing engine.

- Geospatial knowledge provides geospatial knowledge for intelligently processing model integration; it includes a semantics library and a geospatial process chain knowledge library.
- Service repository is a center that accepts the registration of models in a Web service format with semantic annotation.
- Geospatial processing engine is responsible for handling the task from the model consumer.

The purpose of this framework is not only to enable the model owner and the model consumer to perform little work but also to enjoy the model sharing and integration, leaving the sharing and integration challenge to the model broker. To achieve this, some strategies are incorporated in the three core parts of the model broker based on the three phases of the GPW illustrated in Figure 4.

#### 2.2.1. Knowledge Phase

Isolated geospatial models are not designed to be combined together. Geospatial knowledge, including a geospatial process chain knowledge library and a geospatial semantic library, helps to combine models and is prepared by the model broker. A model is regarded here as a process. A process chain (CP) is an ordered logical model combination in semantics. CP is formalized as a tuple as CP = {ID, *N*, *R*, MD}, where ID is the unique identification of the CP, *N* is its name, *R* is the relationship vectors for the models, *R* = {*M*_1_, *M*_2_,…}, *M*_*i*_ presents a model, *M* (mentioned in the beginning of Section 2) with subscript *i* (a finite number), and MD indicates the metadata of the CP. For example, the chlorophyll-a inversion process chain in Figure 4 is CP, the *R* of the CP is *R* = {*M*_1_, *M*_2_, *M*_3_, *M*_4_}, where *M*_1_, *M*_2_, *M*_3_, and *M*_4_ denote the atmospheric correction model, the radiometric correction model, the geometric correction model, and the chlorophyll-a inversion model, respectively. To effectively search a process chain, the semantics library contains ontologies, which formally represents knowledge as a set of concepts within a domain, using shared vocabulary to denote the types, propertie,s and interrelationships of those concepts [51]. A semantics library provides the capabilities of eliminating the inconsistencies among process names and models names. For an application task, the responsibilities of knowledge phase are to find suitable process chains and Web services in three steps: step (1) search the knowledge library to find a suitable process chain for the task, step (2) find suitable Web services for each process in the process chain, and step (3) form service chains of the process chains. In step (1), the semantic terms to describe a task are found or set by model consumer according to the semantics library provided by the model broker. There is a graphical user interface (GUI) developed by the model broker for the model consumer to use when submitting a task. The model broker chooses a task from the ones that have been already listed in the GUI or submits a core term describing the task to the GUI and then finds the task from the returns of the GUI. The process chain library shares the same semantics library. Therefore, the work of step (1) is semantics matching. Step (2) collects all the associated models or Web services of each process and then forms Web service chains of the process chains; this step is automatically completed by the model broker.

#### 2.2.2. Information Phase

The original Web service chains derived from the knowledge phase are mainly semantic chains. The transition from the semantic chains to an executable workflow chain requires Web services composition, data inconsistency processing, and service chain optimization and selection, as shown in Figure 4. 

**Figure 5:**
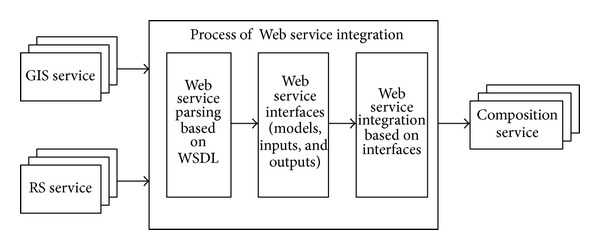
Processes of Web service integration in the model broker.

**Figure 6:**
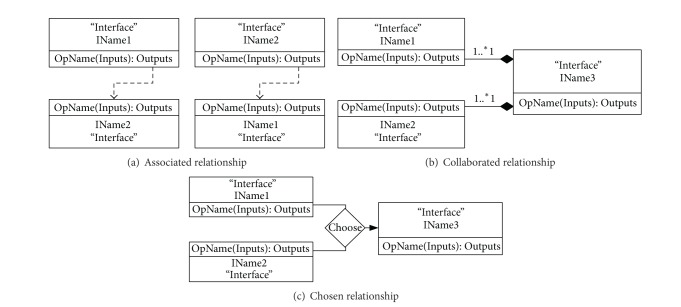
Basic composition relationships between two interfaces.

*Web Services Composition*. The processes of Web service integration are shown in Figure 5. A Web service parsing engine is designed by the model broker to parse RS and GIS Web services with their WSDLs. Next, the Web service interface extracts the appropriate models and composes the models with the WSDL interfaces.  Figure 6 shows three basic composition relationships between two interfaces, interface IName1 and interface IName2, with the pseudo-Unified Modeling Language (UML) diagram. If the output of an operation in an interface is part or all of the input of the other's interface, the two interfaces are associated as shown in Figure 6(a). Their composite is sequential. If the outputs of two interfaces are the inputs of another interface, the two interfaces work together to form a collaborative relationship as shown in Figure 6(b). In contrast with their concurrent appearance, the chosen relationship chooses one interface for a further composite as shown in Figure 6(c). Based on these basic composite relationships, many Web services integrate together for each task. 

**Figure 7:**
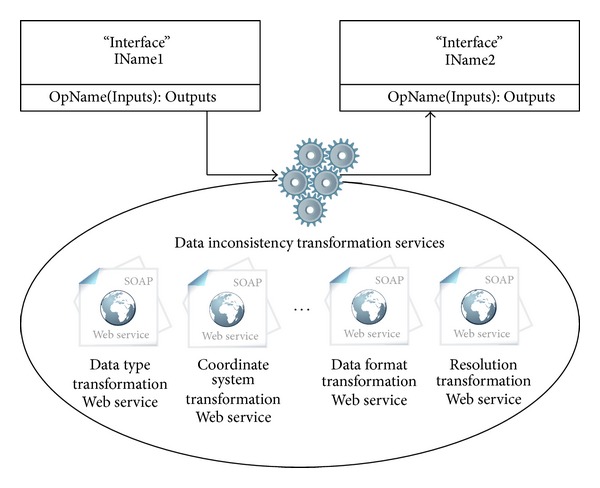
Data inconsistency transformation services.

*Data Inconsistency Processing*. The data flow of the Web service composition is a chain of the inputs and the outputs of the models. The core model executions are black boxes, but the associated models do not need to have consistent data formats. For example, in Figure 6(a), the outputs of IName1 are the logical inputs of IName2, but the formats may not be physically consistent because one is in GoTIFF data format and the other is in IMG data format. The data inconsistency can be caused by the modeler's preference for setting the input and output parameters. Thus, the compositing Web service is not only determining the workflow of the Web services but is also processing the data inconsistencies. Vector and raster data are the two basic and most commonly used data types in GIS and RS. Data inconsistencies of GIS and RS can be external and internal, as shown in Table 1. To overcome this problem, transformation functions are used Web services as shown in Figure 7. Data type transformation, coordinate system transformation, data format transformation, and resolution transformation are necessary transformation functions. These four functions are basic functions in GIS and RS. Widely used professional software includes these functions. For example, the ESRI ArcGIS ArcToolbox provides hundreds of geospatial-related analysis and processing functions, including these four transformation functions. The transformation functions from existing professional software (e.g., ESRI ArcGIS ArcToolbox) have been published into the Web services by the model broker with the method mentioned in Section 2.1 and have been registered in the model broker.

To eliminate data inconsistencies automatically, some rules are defined to register the inputs and the outputs of the functions of a Web service by the model broker. If some parameters of the inputs and the outputs of a function are data, the parameters must be described using their properties: data type (DT), satellite/sensor type (ST), coordinate systems (CS), resolution (RE), and data format (DF). The value of DT is “vector” or “raster.” The ST is the satellite/sensor that obtained the data, such as “MODIS” [52] or “TerraSAR-X” [53]. The representation of CS meets the requirement of the PROJ.4 [54]. RE is numeric. DF is a common data format such as “GeoTiff.” Knowing the five aspects of data inconsistencies, inconsistent data can be transformed to consistent formats. For each model, the model provider describes the five aspects according to the rules defined by model broker.

*Service Chain Optimization and Selection*. To improve efficiency, the integration processes of the Web services need to be optimized and selected. Assume there is a task, TASK_*n*_, that has a series of Web service composition schemes, SCHEME_1_,…,SCHEME_*n*_. The cost of the *i*th (1 ≤ *i* ≤ *n*) scheme is *T*_*i*_. The objective is to determine the scheme with the minimal cost, *Min*⁡(*T*_*i*_). Zeng et al. [55] proposed five generic quality criteria for elementary services: execution price, execution duration, reputation, reliability, and availability; they also selected a global optimal execution scheme. According to the features of this framework, the global optimal execution time is a primary consideration. The final service chain schemes are the outputs of the model broker.

#### 2.2.3. Data Phase

This is a result phase. In this phase, two types of results—data results and workflow schemes results—are provided by the model broker. For the former, a geospatial service chain is executed by execution engine in the model broker to obtain the desired data products. For the latter, the model broker outputs include the workflow schemes described with the service chains, the associated Web services, and the workflow descriptions. The difference between the two is where the workflow is executed: the former is executed at the model broker and the latter is executed at the model consumer. The Business Process Execution Language (BPEL) [56] is an advancing open standards for the information society standard executable language for specifying actions within business processes with Web services. The workflow described using BPEL is flexible and reusable [33, 34]. Therefore, an executable workflow is described using BPEL.

## 3. Example Case and Result

The model sharing and integration method based on Web service that is proposed in this paper is applied for the water environment monitoring in the Pearl River Delta (PRD) region, which is introduced in Section 3.1. Then, the results of this application, including the publication of the model as a Web service and the integration of the models' Web services, are executed, evaluated, and discussed in Sections 3.2 and 3.3.

### 3.1. Pearl River Delta Water Environment Monitoring

The PRD, located between latitudes 21°40′N and 23°N and between longitudes 112°E and 113°20′E, is the low-lying area surrounding the Pearl River estuary in China where the Pearl River flows into the South China Sea. The PRD is a region in China experiencing one of the fastest economy growth rates from China's reformation and opening in 1979. Many large metropolises, such as Guangdong, and the special administrative regions of Shenzhen, Macau, and Hong Kong are nearby. With rapid economic development and urbanization, water environment problems, such as water pollution and water safety, are becoming serious concerns in the PRD [57–60]. To protect the water environment for better living conditions and sustainable development, governments in the PRD area initiated several programs, including developing a water environment monitoring system. Seven research institutes/universities with scholars from a range of scientific domains such as hydrology, ecology, RS, and GIS are collaborating together to develop a water environment monitoring system for the PRD.

Because it is a collaborative effort, an initial problem is that the models are scattered in different areas with distributed systems in the PRD. In addition, some models are not completely open. This is because some models are considered to be core secrets in their institutes and other models are authorization-required and classified. Therefore, some models are not easy to obtain and model owners prefer to provide the “final product” derived from the models rather than the models themselves. In addition, there are different types of models (RS models and GIS models), different running platforms (Linux and Window), and different programming languages/scripts ENVI IDL [61] and PCI EASI [62], which make them difficult to integrate.

**Figure 8:**
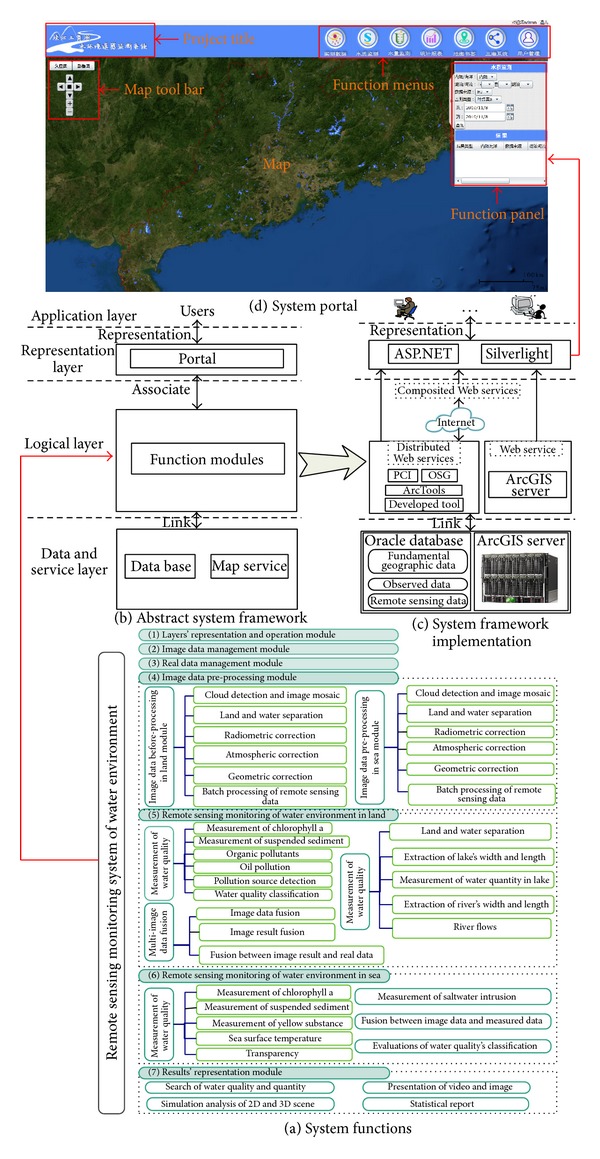
The system functions and system portal.

To overcome the existing problems in the PRD water environment monitoring system, the system functions are performed by sharing and integrating the RS and GIS models based on a Web service, as shown in Figure 8(a). Finally, the water environment monitoring system is developed, including: the functions shown in Figure 8(a), the system framework shown in Figures 8(b)-8(c), and the portal shown in Figure 8(d).

### 3.2. Publishing a Model as a Web Service

The purpose of this experiment is to demonstrate the results of the PRD water environment system using the Web service model sharing method mentioned in Section 2.1. The experiment was performed by codevelopers of the PRD system from several institutes/universities. The codevelopers published their own models using the Web service publishing platform. A subset of the models with their runtime environments are listed as examples in Table 2. The execution methods listed in Table 2 indicate the programming entry of an encapsulated model. From the table, it is evident that models with different development languages and different execution methods are published into the Web services.

### 3.3. Integrating Models as Web Services

The aim of this experiment is to show the models' integration using the Web services. Building geospatial knowledge, managing a task and selecting a reasonable result are each explained. 

*Building Geospatial Knowledge*. Building geospatial knowledge consists of creating the geospatial process chain knowledge library and the geospatial semantics library as shown in Figure 4. The process chains are collected from existing professional software and textbooks. For example, the GIS professional software ArcGIS toolbox has already provided 19 top-level tools with hundreds of functions. RS professional software, ENVI, ERDAS, and PCI also provide hundreds of functions. Then, semantic annotations are made for each process chain. Currently, the semantic annotation of each process in a chain is based on the name and its functional relationship. 

*Managing a Task*. The main work of managing a task is to match a task with a process and its Web services. The semantic match of a task with a process chain is based on the semantic term. For example, if a task is to calculate chlorophyll a, then the task with the semantic term “chlorophyll a” and the process chain with semantic term “chlorophyll a” are matched. Because the semantics and the Web services of a process are registered with fixed standards at the beginning by the model providers, the suitable Web services for a process chain will be found. The data inconsistency processing includes transforming data to another data format (DT, ST, CS, RE, and DF). 

**Figure 9:**
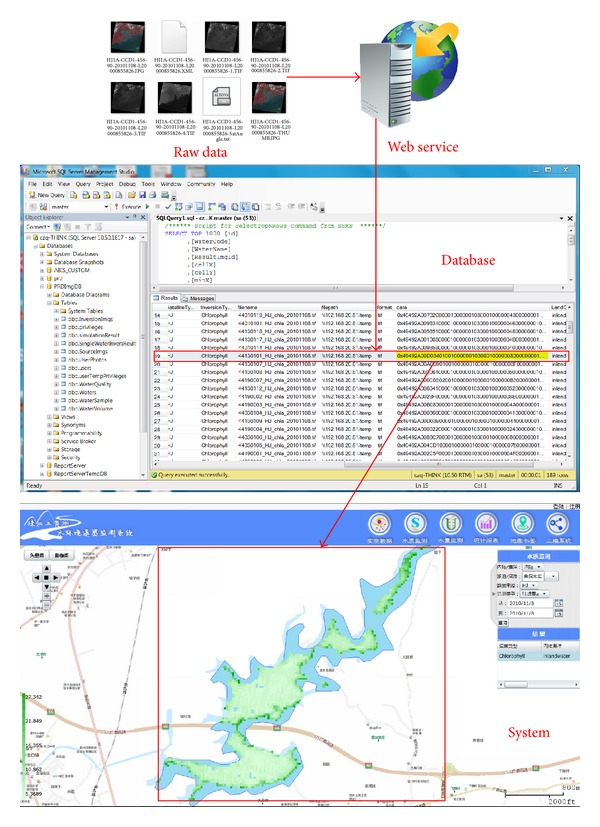
The result of scheme 1.

*Selecting a Reasonable Service Chain*. The result of the model broker is a data result or a set of model integration schemes. In the PRD system, the model integration schemes are chosen. The model broker shows the reasonable results as a list, for example, a scheme for separating land and water is introduced. Separating land and water is a critical step for extracting water area and the water environment. The raw data are processed radiometric correction, atmospheric correction, and image segmentation to separate land and water. Two resulting schemes are return from the model broker, and two examples are tested. The data in example 1 is a 259 MB TM image; the data in example 2 is an 826 MB HJ image. The time costs of the two examples are provided in Table 3. As shown in Table 3, the costs of schemes 1 and 2 are 82.44% and 44.43%, respectively. Therefore, the efficiency of scheme 1 is higher, and scheme 1 is more highly recommended than scheme 2. The result of scheme 1 is shown in Figure 9.

## 4. Discussion and Conclusion

Sharing and integrating RS and GIS models are important for their application. This paper studies the framework of sharing and integrating RS and GIS models using a Web service. In the framework, a black box method with a visual interface is proposed for rapid Web service publishing. This method will facilitate the publication of a model to the Web service; in addition, it will assist researchers in concentrating their efforts on model development rather than on programming. In addition, integrating workflow and using semantics supported method is an effective way to integrate models using Web services. The framework is applied in the development of the PRD water environment monitoring system in which RS and GIS models are integrated.

The advanced features of this framework are facilitating the model provider's and model consumer's work of model sharing and integration and integrating the workflow-based service composition method and the AI-based service composition method. For the former, the framework provides the model provider and the model consumer methods for rapidly publishing and integrating their models. A model provider can publish a model using a visual interface, and a model consumer chooses the semantic terms of the task. This frees the model providers and the model consumers from tedious work and improves their work efficiency. For the latter, the framework integrates the workflow-based service composition method and the AI-based service composition method, making model integration intelligent, automatic, and industrialized.

Further studies will focus on the following aspects:

- enhancing and enriching the geospatial knowledge: the geospatial knowledge in the model broker is derived from professional software and textbooks. The process chains and semantic information are limited, requiring enhancement and enrichment to meet the requirement of broader applications.
- improving the automatic processing ability: the automatic processing ability depends on the geospatial knowledge and the Web services composition. Currently, the Web services composition is associated with the process chains. More powerful and efficient association between a process chain and its Web services require additional work.

## Figures and Tables

**Table 1:** Data inconsistency of two data types.

Data 1	Data 2	Presentation of data inconsistency
Vector data	Raster data	Data type
Raster data	Vector data	Data type
Vector data	Vector data	Coordinate system
Raster data	Raster data	Coordinate system Resolution Data format

**Table 2:** Models and their runtime environments.

Model name	Platform	Language	Execution method
Atmospheric correction model	Microsoft Windows	ENVI IDL	ENVI script (.pro)
Chlorophyll-a inversion model	Microsoft Windows	PCI IDL	PCI script (.eas)
Projection transformation model	Microsoft Windows	C#	EXE

**Table 3:** The cost comparisons of two Web services methods.

Scheme name	Cost (second)	
	Example 1	Example 2
Scheme 1	534.840	1012.558
Scheme 2	648.795	2278.76
Ratio	82.44%	44.43%
